# The Effect of Fluoxetine on Progression in Progressive Multiple Sclerosis: A Double-Blind, Randomized, Placebo-Controlled Trial

**DOI:** 10.1155/2013/370943

**Published:** 2013-07-29

**Authors:** Jop Mostert, Thea Heersema, Manju Mahajan, Jeroen Van Der Grond, Mark A. Van Buchem, Jacques De Keyser

**Affiliations:** ^1^Department of Neurology, University Medical Center Groningen, University of Groningen, Postbus 30.001, 9700 RB Groningen, The Netherlands; ^2^Department of Neurology, Rijnstate Hospital, Postbus 9555, 6800 TA Arnhem, The Netherlands; ^3^Department of Radiology, Leiden University Medical Center, Albinusdreef 2, 2333 ZA Leiden, The Netherlands; ^4^Department of Neurology, Center for Neurosciences, University Hospital Brussels, Vrije Universiteit Brussel (VUB), Laarbeeklaan 101, 1090 Brussels, Belgium

## Abstract

Preclinical studies suggest that fluoxetine may have neuroprotective properties. In this pilot study forty-two patients with secondary or primary progressive MS were randomized to receive fluoxetine 20 mg twice daily or placebo for 2 years. Every 3 months the Expanded Disability Status Scale (EDSS), 9-hole peg test (9-HPT) and ambulation index (AI) were assessed. Brain MRI scans, Multiple Sclerosis Functional Composite, Fatigue Impact Scale, Guy's neurological disability Scale and SF-36 were performed at baseline, year 1 and year 2. Seven out of 20 (35%) patients in the fluoxetine group and 7 out of 22 (32%) patients in the placebo group had sustained progression on the EDSS, 9-HPT, or AI at 2 years. No differences were identified between the 2 treatment groups with respect to secondary clinical outcomes and T2 lesion load, grey matter volume and white matter volume. An unanticipated low rate of disability progression in the placebo group decreased the statistical power. At least 200 patients would have been needed to detect a 50% treatment effect. This trial shows that fluoxetine was generally well tolerated, but no assumptions can be made about a possible treatment effect. An adequately powered controlled trial of fluoxetine in progressive MS is still warranted. This trial is registered with Current Controlled Trials ISRCTN38456328.

## 1. Introduction

The progressive phase of multiple sclerosis (MS) reflects a poorly understood insidious axonal degeneration that is age related and independent of relapses [1]. Currently available disease-modifying treatments, which act by modifying the immune response, are largely ineffective in progressive MS [2–4].

A reduced axonal energy metabolism, glutamate toxicity, and decreased brain-derived neurotrophic factor (BDNF) levels are suspected to be involved in the widespread axonal degeneration that underlies progression in progressive MS [5–7]. Astrocytes in MS appear to be deficient in *β*_2_-adrenergic receptors that are involved in astrocytic glycogenolysis necessary for the maintenance of the sodium dependent glutamate uptake and for the release of lactate, which is an energy source for axons [8–11]. 

Fluoxetine, a selective serotonin-reuptake inhibitor (SSRI), might be able to protect against axonal loss underlying the progressive phase of MS because it stimulates glycogenolysis and it enhances the production of brain-derived neurotrophic factor in rodent astrocyte cultures [12, 13]. 

After 2 weeks of treatment with fluoxetine (first week 20 mg/day and second week 40 mg/day) a significantly improved cerebral white matter NAA/creatine ratio was found on MRI, suggesting an improvement in axonal mitochondrial energy metabolism [14]. Fluoxetine might also suppress the antigen-presenting capacity of glial cells, and a pilot study in patients with MS with relapses found that a daily dose of 20 mg tended to reduce the formation of new inflammatory lesions [15]. 

Based on these preliminary findings and theoretical benefits, we decided to perform a pilot study to assess whether fluoxetine is well tolerated and might have a neuroprotective effect in patients with progressive MS.

## 2. Methods

### 2.1. Patients

The local medical ethics committee approved the protocol, and all patients provided written informed consent. Eligible patients were 18 to 65 years of age and had primary or secondary progressive MS according to the revised McDonalds' criteria [16]. Additional inclusion criteria were an Expanded Disability Status Score (EDSS) of 3.5 through 6.5 [17] and documented progression in the two years preceding the study unrelated to clinical relapse. Exclusion criteria were the use of immunomodulatory, immunosuppressive, or antidepressants drugs or lithium in the previous 6 months, the use of corticosteroids in the 3 months prior to start of the study, depression defined as a score of 19 or higher on Beck's Depression Inventory II [18], bipolar disorder, contraindication to magnetic resonance imaging (MRI), other neurological or systemic disorder that would interfere with the assessments, and pregnancy or unwillingness to use acceptable birth control.

### 2.2. Study Design

This single-center, double-blind, placebo-controlled study was initiated in 2005. Patients were randomized 1 : 1 to fluoxetine or placebo and were stratified according to their disease course. During the first 2 weeks patients used one tablet and thereafter two tablets of fluoxetine 20 mg or identical placebo daily for a total duration of 2 years. After a screening visit prior to start of the study medication, the MS Functional Composite (MSFC) [19], Expanded Disability Status Scale (EDSS), and ambulation index (AI) [20] were performed at baseline and every 3 months for 2 years. At baseline, year 1, and year 2 the Fatigue Impact Scale (FIS) [21], Guy's Neurological Disability Scale (GDNS) [22], and SF-36 [23] were carried out. Two physicians (DH and JM) were responsible for enrollment of participants and EDSS assessments. The other clinical scales were assessed by nurse practitioners. MRIs were performed at baseline, year 1, and at the end of the study. 

The hospital pharmacy produced the tablets of fluoxetine 20 mg and placebo of identical appearance and performed the randomization. Only the pharmacist was aware of treatment allocation throughout the study. The randomization code was revealed to the researchers after all analyses were completed. 

### 2.3. MRI Protocol and Analysis

All scans were performed on a 3.0 Tesla scanner (Philips) with an eight-channel SENSE head coil. Brain transaxial Dual TSE (repetition time, 3000 msec; echo times, 27 and 120 msec; slice thickness, 3 mm), FLAIR (repetition time, 11,000 msec; echo time, 100 msec; slice thickness, 3 mm), and 3D High Resolution T1-weighted (repetition time, 7.5 msec; echo time, the shortest) images were obtained at baseline, year 1, and year 2. 

The scans were blindly analyzed at the Department of Radiology of the Leiden University Medical Center. To calculate normalized grey and white matter volumes, all T1-weighed scans were analyzed using software FMRIB's Automated Segmentation Tool (FAST) provided by FMRIB's Software Library (FSL) [24]. Total grey and white matter tissue volumes were estimated with SIENAX. This program extracts brain and skull images from the single whole-head input data. The brain images were then affine-registered to MNI152 space, using the skull images to determine the registration scaling. Next, tissue-type segmentation with partial volume estimation was carried out in order to calculate total volumes of brain tissue. 

T2 lesion load (T2LL) was assessed semiautomatically, using Software for Neuro-Image Processing in Experimental Research (SNIPER), an in-house developed program for image processing [25]. T2LL volumes were normalized according to the scaling factor obtained by the T1 registration to MNI152 in FSL.

### 2.4. Outcome Measures

The primary outcome measure was the number of patients with progression in 2 years. Progression was defined as either worsening of EDSS of 1.0 point or more for a baseline EDDS of 3.0 to 5.0 or 0.5 point or more for a baseline EDSS of 5.5–6.5, a worsening of 9-hole peg test (9-HPT) of more than 20% compared to the baseline 9-HPT, or an increase of 1.0 or more of the AI when the baseline score was between 2.0 and 6.0. Progression needed to be confirmed at two follow-up assessments and at the end of study. 

Secondary clinical endpoints were changes in EDSS, MSFC, FIS, GNDS, and SF-36. The MSFC is a multidimensional test consisting of a task for leg function (timed 25-foot walk), arm function (9-hole peg test), and cognition (paced auditory serial addition test) [19]. Its score represents the mean of the z-scores of the three tests, which are calculated in comparison to a pooled reference population [26]. Lower scores indicate more disability. The FIS is a questionnaire evaluating fatigue with higher scores indicating more complaints [21]. With the GDNS [22] and SF-36 [23] patients are self-reporting their neurological and functional disability in a variety of domains.

MRI outcomes included change in T2 lesion load (T2LL), change in white matter volume, and change in grey matter volume. 

When patients were lost to followup, the last observation was used in the analysis.

### 2.5. Statistics

We estimated sample size on a study assessing the effect of methotrexate on progression of disability in patients with progressive MS [27]. In this study, using a comparable definition of progression of disability, 80% of the placebo group encountered progression of disability during 2-year followup. We estimated that we needed 26 patients per treatment arm with a power of 0.8 to detect a 50% reduction of progression of disability with fluoxetine. Expecting a 10% dropout rate, we planned to include 30 patients per treatment arm.

All data were tested for normality. To determine the effect of fluoxetine on time to progression, we performed Cox proportional-hazards regression analyses to calculate hazard ratios with adjustment for age, disease duration, disease course, and gender. 

Baseline and follow-up data were evaluated with the independent sample *t*-test or Wilcoxon-Mann-Whitney rank-sum test when appropriate. The *χ* ^2^ test and Fisher's exact test were used to compare differences in categorical variables. Analyses were performed with the Statistical Package for the Social Sciences (SPSS 16.0 for Windows, Chicago, IL, USA). All reported *P* values are two-tailed. Significance was taken at 0.05.

## 3. Results

### 3.1. Patients

**Figure 1:**
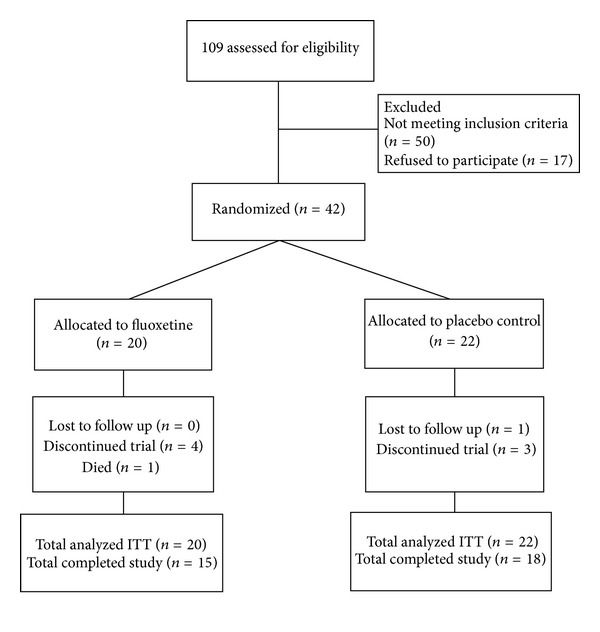
Flow of the patients.

Of 109 patients screened, 42 were randomized to fluoxetine (*n* = 20) or placebo (*n* = 22). Inclusion started in 2006 and was stopped in October 2008. Inclusion was slow and we had to terminate the study prematurely because of the expiration date of the study medication.  Figure 1 shows the flow of the patients.

Baseline characteristics were comparable between patients receiving fluoxetine and placebo (Table 1). Five patients (3 due to side effects, 1 due to progression of disability, and 1 deceased due to myocardial infarction) using fluoxetine and 4 patients (3 due to side effects and 1 moved to another town) using placebo did not complete the study. The patient who died from myocardial infarction 19 months after starting the study medication was a heavy cigarette smoker for 35 years.

### 3.2. Effect of Fluoxetine on Progression

Seven patients using fluoxetine and 7 patients using placebo had progression of disability during 2 years of treatment. The progression of disability was most often established on the EDSS. For details see Table 2.

A Cox regression analysis (Table 3) showed no effect of fluoxetine on time to progression. 

### 3.3. Effect of Fluoxetine on EDSS, MSFC, FIS, GNDS, and SF-36

There was no difference in the change in EDSS, MSFC, FIS, and GNDS between patients using fluoxetine and placebo (Table 4). Changes in all SF-36 domains were also comparable (data not shown). 

### 3.4. Effect of Fluoxetine on MRI Outcomes

There was no difference in the increase of T2LL. The decreases in grey matter and white matter volumes were also comparable (Table 5).

### 3.5. Side Effects

There was one myocardial infarction in the fluoxetine group. Since the use of SSRIs is associated with a slightly decreased risk of myocardial infarction, this is most likely not related to the use of the study medication [28]. There were no other serious adverse events. Patients using fluoxetine more often suffered from drowsiness and fatigue, which was mainly at the start of treatment (Table 6).

## 4. Discussion

This study showed no effect of fluoxetine on progression of disability in patients with progressive MS. Compared to placebo, patients using fluoxetine suffered more often from drowsiness and fatigue, but in general fluoxetine taken at a dose of 40 mg daily was well tolerated. 

Inclusion was slow, especially because the frequent use of SSRIs and tricyclic antidepressants excluded participation of many patients. Because the study medication expiry date was reached, the study was discontinued before inclusion was complete. 

We did not find a difference between fluoxetine and placebo on progression of disability and secondary clinical and MRI outcomes. However, our assumptions regarding progression rate of the placebo treated patients proved to be incorrect. We based our sample size on the methotrexate study in patients with progressive MS [27]. In that 2-year study 80% of the placebo group encountered progression of disability, using a similar composite score of progression as in our trial. A more recent study in 161 patients with primary progressive MS found that after 2 years, 63% had progressed on either the EDSS, T25FW, or 9HPT [29]. In our study only 32% of the patients in the placebo group had progressed over 2 years. This means that with a sample size of 25 patients in each group we would have been only able to detect a more than 90% decrease in the proportion of patients that progresses with an 80% power and a significance level of 0.05. To detect a treatment effect of 50%, we would have needed 100 patients per treatment arm. In the glatiramer acetate in primary progressive MS trial a similar low rate of progression based on the EDSS over 2 years was found in the placebo group as in our study [3]. These data were not available at the time that we started our trial. 

There are increasing data about possible neuroprotective and neuroregenerative effects of fluoxetine on animal models [30–32]. In patients with ischemic stroke the early use of 20 mg fluoxetine with physiotherapy enhanced motor recovery after 3 months [33].

## 5. Conclusion

In this underpowered study no effect of fluoxetine on progression of disability was found. An adequately powered controlled trial of fluoxetine in progressive MS is still warranted and should at least include 100 patients per treatment arm. 

## Figures and Tables

**Table 1:** Baseline characteristics.

	Fluoxetine	Placebo	*P* value
Number	20	22	
Disease course (SPMS/PPMS)	14/6	15/7	0.90
Gender (male/Female)	12/8	12/10	0.72
Age (years; mean, sd)	49.7 (9.2)	47.5 (7.6)	0.42
Disease duration (years; mean, sd)	14.8 (9.0)	13.0 (6.2)	0.45
EDSS (median, IQR)	6.0 (5.0–6.5)	5.75 (4.0–6.5)	0.90
MSFC (mean, sd)	−0.29 (0.84)	−0.33 (0.44)	0.87
9HPT, sec (mean, sd)	36 (33)	30 (10)	0.36
AI (median, IQR)	3.5 (2.0–4.75)	2.0 (2.0–5.0)	0.47
FIS	42 (29)	44 (32)	0.82
GNDS	12 (7)	11 (4)	0.53
BDI	9 (7)	10 (6)	0.50
T2LL (ml; mean, sd)	7.8 (8.8)	9.9 (9.1)	0.47
WM volume (ml; mean, sd)	624.5 (51.8)	636.4 (56.2)	0.48
GM volume (ml; mean, sd)	622.6 (58.9)	632.4 (57.9)	0.59

EDSS: Expanded Disability Status Scale; MSFC: Multiple Sclerosis Functional Composite; 9HPT: 9-hole peg test; AI: ambulation index; FIS: Fatigue Impact Scale; GDNS: Guy's Neurological Disability Scale; BDI: Beck's Depression Inventory II; T2LL: T2 lesion load; WM: White Matter; GM: Grey Matter.

**Table 2:** Number of patients with progression by disease course.

	Fluoxetine	Placebo
All (*N*)	20	22
Progression	7 (35%)	7 (32%)
Time to progression (months; mean, sd)	7.7 (5.2)	10.7 (6.4)
EDSS progression	5 (25%)	7 (32%)
9HPT progression	1 (5%)	3 (14%)
AI progression	2 (10%)	1 (4.5%)

SPMS (*N*)	14	15
Progression SPMS	5 (36%)	5 (33%)
Time to progression (months; mean, sd)	9.0 (5.6)	12.0 (6.7)
EDSS progression SPMS	3 (21%)	5 (33%)
9HPT progression SPMS	1 (7%)	2 (13%)
AI progression SPMS	1 (7%)	1 (6.7%)

PPMS (*N*)	6	7
Progression PPMS	2 (33%)	2 (29%)
Time to progression (months; mean, sd)	4.5 (2.1)	7.5 (6.4)
EDSS progression PPMS	2 (33%)	2 (29%)
9HPT progression PPMS	0 (0%)	1 (14%)
AI progression PPMS	1 (17%)	0 (0%)

EDSS: Expanded Disability Status Scale; 9HPT: 9-hole peg test; AI: ambulation index.

**Table 3:** Cox regression analyses of time to progression by treatment group controlled for gender, disease course, age and disease duration.

		Reference	HR (95% CI)	*P* value
Treatment	Placebo	1.00		
	Fluoxetine		1.15 (0.38–3.43)	0.81
Gender	Male	1.00		
	Female		2.96 (0.96–9.10)	0.06
Disease course	PPMS	1.00		
	SPMS		1.08 (0.25–4.64)	0.92
Age	Per year increase		1.05 (0.96–1.15)	0.26
Disease duration	Per year increase		1.00 (0.92–1.08)	0.94

**Table 4:** Change in clinical scores.

	Fluoxetine	Placebo	*P* value
Change in EDSS^#^ (median, range)	0.0 (−0.5–3.5)	0.0 (−1.0–2.0)	0.56
(mean, sd)	0.38 (0.86)	0.20 (0.68)	
Change in MSFC^#^ (mean, sd)	−0.41 (1.19)	−0.10 (1.00)	0.36
Change in FIS* (mean, sd)	−2.7 (16)	−3.3 (33)	0.95
Change in GNDS* (mean, sd)	1.3 (4)	0.7 (5)	0.76

^#^Fluoxetine = 20, placebo = 22; *fluoxetine = 16, placebo = 19;

EDSS: Expanded Disability Status Scale; MSFC: Multiple Sclerosis Functional Composite; FIS: Fatigue Impact Scale; GDNS: Guy's Neurological Disability Scale.

**Table 5:** MRI outcomes (mean, sd).

	Fluoxetine (*N* = 16)	Placebo (*N* = 19)	*P* value
Change in T2LL (mL)	0.11 (0.3)	0.31 (2.9)	0.80
Change in WM volume (mL)	−28.9 (68.7)	−16.9 (62.6)	0.59
Change in GM volume (mL)	−37.7 (68.7)	−18.7 (55.6)	0.37

T2LL: T2 lesion load; WM: white matter; GM: grey matter.

**Table 6:** Side effects.

	Fluoxetine (*n* = 20)	Placebo (*n* = 22)	*P* value
Headache	5	4	0.65
Dizziness	8	5	0.27
Nausea*	3	3	0.95
Drowsiness*	9	2	0.01
Fatigue*	4	0	0.03
Hyperhidrosis	2	0	0.13
Reflux esofagitis	2	0	0.13

*Mainly at the beginning of the study.
